# Insufficient Iodine Level in Urine among Children of a Secondary School: A Descriptive Cross-sectional Study

**DOI:** 10.31729/jnma.6084

**Published:** 2021-10-31

**Authors:** Anand Ballabh Joshi, Megha Raj Banjara, Chitra Kumar Gurung, Vivek Kumar Singh, Krishna Pant, Chikayoshi Atsuta, Aditya Joshi

**Affiliations:** 1 Public Health and Infectious Disease Research Center, Kathmandu, Nepal; 2 Central Department of Microbiology, Tribhuvan University, Kirtipur, Nepal; 3 Society for Eliminating Nepalese Iodine Deficiency (Senid), Osaka, Japan

**Keywords:** *iodine*, *salt*, *urine*

## Abstract

**Introduction:**

Iodine deficiency disorders are common endocrinopathies in Nepal. Children are at risk for iodine deficiency disorders. Irreversible mental retardation and brain damage in children are the devastating disorders lead by iodine deficiency. Therefore, the main objective of the study was to find out the prevalence of insufficient iodine level among the children of a secondary school.

**Methods:**

This descriptive cross-sectional study was conducted in urine from April 2019 to July 2019 after obtaining ethical approval from Nepal Health Research Council (Registration number: 802/2018). Forty-six urine samples were collected from school children for iodine estimation. Convenience sampling was done. Data were entered into Statistical Package for the Social Science version 21 and descriptive analyses were done. Point estimate at 95% confidence interval was calculated along with frequency and proportion for binary data.

**Results:**

Among the 46 students, majority 36 (78.30%) (95% Confidence Interval= 66.30-90.21) of the school children had insufficient urine iodine level. Among 36 salt samples collected from school children with low urine iodine level, 8 (22.2%) salt samples had iodine less than 15ppm.

**Conclusions:**

Iodine estimation revealed a very high percentage of urine samples containing insufficient levels of iodine is similar as compared to studies done in similar settings. Hence, the school children were at risk of iodine deficiency disorders. Iodine deficiency disorders prevention programs should be priority intervention based on available evidence.

## INTRODUCTION

An array of morbidities resulting from the micronutrient iodine is termed iodine deficiency disorder (IDD).^1,2^ Most devastating consequences of iodine deficiency are the impaired growth and neurodevelopment of the offspring.^3^,^4^ Iodine, helps to synthesize the hormone thyroxine and its daily requirement is infinitesimally small (150-200|μg).^5^"^8^

The most prominent and cost effective strategy adopted by majority countries to control iodine deficiency is through universal salt iodization.^3^ In 1973, to address iodine deficiency disorder, the Ministry of Health and Population, Nepal, adopted a policy to fortify all edible salt through universal salt iodization. The households using adequately iodized salt have increased from 55% in 1998 to 95% in 2016 as reported by the national survey. However, disparities persist in the use of iodized salt.^9^

Therefore, the main objective of the study was to find out the prevalence of insufficient iodine level among the children of the secondary school.

## METHODS

This was a descriptive cross-sectional study conducted among school children in Ramadevi Secondary School in Sanga Chowk, Sindhupalchowk district, Nepal in the year 2019 from April to July. Ethical approval was obtained from the Nepal Health Research Council (Registration number: 802/2018). The school children agreeing to give informed consent were included in the study while those refusing for informed consent were excluded from the study. School children were selected through a convenience sampling method. Informed consent was obtained from all the participants.

In a cross-sectional study, the proportion of school children with inadequate urine iodine level was 11.1%.^10^

The sample size was calculated by using formula,

n = Z^2^ × p × q / e^2^

  = (1.96)^2^ × (0.111) × (0.889) / (0.1)^2^

  = 38

Where,

n= required sample size
Z = 1.96 at 95% Confidence Interval (CI)
p = prevalence from the previous study, 11.1%^10^
q = 1-p
e = margin of error, 10%

Taking a non-response rate of 10%, the calculated sample size is 42. However, a sample size of 46 was taken.

Forty-six secondary level school children (class 9 and 10) from the school were selected for the study. Urine samples were collected from the school children.

Urine samples were collected from school children for the estimation of urine iodine to screen IDD status. Samples were collected in clean leak proof containers and refrigerated at -20°C until analysis. The urinary iodine excretion (UIE) analysis was conducted at Biochemistry Department, BP Koirala Institute of Health Sciences (BPKIHS), Dharan.

The urine and quality control (QC) specimens were allowed to reach the ambient temperature. The sample was vortexed well before taking an aliquot for analysis such that no particles remain on the bottom of the tube. Two hundred fifty μl of each urine sample, working standards and bench QC was pipetted into a 13 x 100mm test tube. All samples were pipetted in duplicate. One ml of ammonium persulfate solution was added to each tube. All the solutions were mixed and heated on a heating block for 60 minutes at 91-95°C (digestion step). After digestion, the tubes were cooled to room temperature. Arsenious acid solution (3.5ml) was added, mixed and left to stand for 15minutes. Ceric ammonium sulfate solution (400μl) was added to each tube and quickly vortexed to mix (A timer was used to keep a constant interval of 30 seconds between additions to successive tubes). Exactly 30 minutes after the addition of ceric ammonium sulfate to the first tube, the absorbance was noted at 420nm in a spectrophotometer. The successive tubes were read at the same time intervals as when adding the ceric ammonium sulfate.

Salt samples were collected from children with low urine iodine level in an air-tight container and were transported to the laboratory for iodine estimation through titration method.

Collected data were entered in Statistical Package for Social Sciences version 21 and analyzed for risk factors and outcome variables. Descriptive analyses were presented and analyzed. The classification of urinary iodine excretion (UIE) for school-age children was used as per WHO guidelines.^11^

## RESULTS

**Figure 1:**
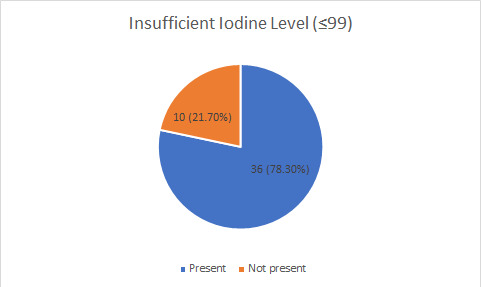
Urinary iodine level among students.

Among the 46 students, 36 (78.30%) (66.30-90.21 at 95% Confidence Interval) of the school children had insufficient urine iodine level (Figure 1).

Among 36 salt samples collected from school children with insufficient urine iodine level, 8 (22.2%) salt samples had iodine less than or 15ppm whereas 28 (77.8%) had more than 15ppm iodine. Mean of iodine in 36 salt samples was 34.75ppm and standard deviation was 19.56ppm (Table 1).

**Table 1:** Salt iodine level among children with insufficient urine iodine levels (n= 36).

UIE level (μg/l)	Salt iodine level (ppm)		
	≤15 n (%)	>15 n (%)	Total n (%)
Insufficient (<150)	8 (22.2)	28 (77.8)	36 (78.3)

## DISCUSSION

The preventable cause of morbidities in children resulting from the iodine deficiency is still a threat to the Nepalese population. Despite the achievements of Ministry of Health, Department of Health services and salt trading corporation through salt iodization and community awareness about the use of iodized salt through mass communication; several researchers have reported discrepancies in urine iodine of school children,^10,12,16^ and in iodine content of household salts^17,19^ from different geographical areas of the country.

In our study, the insufficient iodine level was found to be 78.30% which was high compared to the study conducted by Kunwar, et al.^10^ In a study conducted in terai region by Khatiwada, et al. 12.7% children were iodine deficient and 34.2% had excessive iodine level.^13^ To overcome the effects of iodine deficiency disorder in children, Government of Nepal started a campaign to fortify all edible salts.^9^ Besides, tremendous effort has been made to make people aware about the iodized salt through radios and televisions. Such activities have created a good impact on the Nepalese people as some research has found above 95% households using iodized salt.^20^ Despite all these efforts, iodine deficiency is being reported in this study.

This study found 77.8% households salt contained more than 15ppm iodine whereas Khatiwada, et al. reported only 9.4% sample had iodine content less than 15ppm.^17^ The iodized salt must contain 15ppm iodine up to the household level, however 22.2% households salts in this study did not contained appropriate iodine level. This might be the point of concern as it may be a factor contributing to the low iodine levels in school children. Hence, there is a need for a study to investigate the reasons of inadequacy of iodine level in the iodized salt which might answer the cause of iodine deficiency in children when they are consuming iodized salts.

This study was limited with the low sample size because of limited funding. The findings of this study can't be generalized as this is conducted in a single setting. Also, being a descriptive cross-sectional study, association between the variables cannot be shown.

## CONCLUSIONS

Iodine estimation revealed a very high percentage of urine samples containing insufficient levels of iodine is similar as compared to studies done in similar settings. IDDs prevention program should be priority intervention based on available evidence.
